# MRI/RNA-Seq-Based Radiogenomics and Artificial Intelligence for More Accurate Staging of Muscle-Invasive Bladder Cancer

**DOI:** 10.3390/ijms25010088

**Published:** 2023-12-20

**Authors:** Touseef Ahmad Qureshi, Xingyu Chen, Yibin Xie, Kaoru Murakami, Toru Sakatani, Yuki Kita, Takashi Kobayashi, Makito Miyake, Simon R. V. Knott, Debiao Li, Charles J. Rosser, Hideki Furuya

**Affiliations:** 1 Biomedical Imaging Research Institute, Cedars-Sinai Medical Center, Los Angeles, CA 90048, USA; touseefahmad.qureshi@cshs.org (T.A.Q.); yibin.xie@cshs.org (Y.X.); debiao.li@cshs.org (D.L.); 2 Department of Biomedical Science, Cedars-Sinai Medical Center, Los Angeles, CA 90048, USA; xingyu.chen@cshs.org (X.C.); simon.knott@cshs.org (S.R.V.K.); 3 Department of Urology, Cedars-Sinai Medical Center, Los Angeles, CA 90048, USA; charles.rosser@cshs.org; 4 Samuel Oschin Comprehensive Cancer Institute, Cedars-Sinai Medical Center, Los Angeles, CA 90048, USA; kaorum@kuhp.kyoto-u.ac.jp (K.M.); toru.sakatani@cshs.org (T.S.); 5 Department of Urology, Kyoto University, Kyoto 606-8507, Japan; kitayuki@kuhp.kyoto-u.ac.jp (Y.K.); selecao@kuhp.kyoto-u.ac.jp (T.K.); 6 Department of Urology, Nara Medical University, Kashihara 634-8522, Japan; makitomiyake@yahoo.co.jp

**Keywords:** bladder cancer, radiogenomics, artificial intelligence, MRI, RNA-seq

## Abstract

Accurate staging of bladder cancer assists in identifying optimal treatment (e.g., transurethral resection vs. radical cystectomy vs. bladder preservation). However, currently, about one-third of patients are over-staged and one-third are under-staged. There is a pressing need for a more accurate staging modality to evaluate patients with bladder cancer to assist clinical decision-making. We hypothesize that MRI/RNA-seq-based radiogenomics and artificial intelligence can more accurately stage bladder cancer. A total of 40 magnetic resonance imaging (MRI) and matched formalin-fixed paraffin-embedded (FFPE) tissues were available for analysis. Twenty-eight (28) MRI and their matched FFPE tissues were available for training analysis, and 12 matched MRI and FFPE tissues were used for validation. FFPE samples were subjected to bulk RNA-seq, followed by bioinformatics analysis. In the radiomics, several hundred image-based features from bladder tumors in MRI were extracted and analyzed. Overall, the model obtained mean sensitivity, specificity, and accuracy of 94%, 88%, and 92%, respectively, in differentiating intra- vs. extra-bladder cancer. The proposed model demonstrated improvement in the three matrices by 17%, 33%, and 25% and 17%, 16%, and 17% as compared to the genetic- and radiomic-based models alone, respectively. The radiogenomics of bladder cancer provides insight into discriminative features capable of more accurately staging bladder cancer. Additional studies are underway.

## 1. Introduction

Bladder cancer is the fourth and twelfth most common cancer in men and women, respectively, in the United States. An estimated 82,290 newly diagnosed cases of bladder cancer and 16,710 deaths from bladder cancer are expected to occur in 2023 [1]. Bladder cancer has one of the highest recurrence rates of any tumor type [2]. When diagnosed early as a T0/T1 lesion or even a T2 lesion, a cure with surgical resection is possible in a high percentage of cases, with a 5-year survival rate of >94% and >50%, respectively [3,4]. However, once the tumor extends beyond the muscle lining of the bladder, the 5-year survival rate is <50%, while metastatic disease is almost always fatal, with an estimated median survival of 12 to 14 months and a 5-year survival of <20% [5]. Muscle-invasive bladder cancer (MIBC) comprises approximately one-third of bladder cancer and is associated with significant morbidity and mortality. The treatment of patients with MIBC has traditionally been managed through radical cystectomy [6]. However, radical cystectomy is accompanied by significant treatment morbidity and mortality, as well as a substantive change in the quality of life associated with the removal of the urinary bladder [7]. On the other hand, studies have shown that for highly select patients, the outcomes of bladder preservation with chemoradiotherapy may offer comparable survival outcomes to those of radical cystectomy [6,8,9]. Therefore, it is important to properly select the patients suitable for the bladder-preserving strategy. Currently, a robust staging modality (i.e., determining the extent of cancer) is lacking, with approximately one-third of patients being under-staged and one-third of patients being over-staged with axial imaging of the abdomen and pelvis with computed tomography (CT) imaging [10]. Thus, accurate staging of bladder cancer is essential to identifying the optimal treatment.

In-depth investigation of genetic and tumor-imaging information associated with different stages of bladder cancer may provide insight into unique patterns that could efficiently assist in distinguishing early-stage (i.e., intra-vesical) from late-stage (i.e., extra-vesical) cancers. Validating reliable biomarkers for MIBC staging remains an ongoing challenge [11], as bladder cancer is known for its molecular and clinical heterogeneity, posing challenges in developing a universally applicable staging system [12]. Clinical and pathological staging through transurethral resection of bladder tumors (TURBT) is prone to inter-observer variability, affecting the consistency of staging results [13]. Incorporating imaging modalities and genomic profiling can be advantageous but presents challenges in terms of integration, interpretation, and standardization [14,15].

Fortunately, artificial intelligence (AI) offers numerous tools and techniques to thoroughly examine genomic and imaging data, as well as the integration of the genomic and imaging data with the expressed purpose of improving the accuracy of current staging [16,17]. In recent years, a range of automated analysis and modeling techniques have been developed, particularly for genomic and radiomic data, including tools for extracting precise measurements of biomarkers and organs, unveiling complex features, and quantifying tissue characteristics [18,19,20]. The advancements in radiomic, machine learning, and deep learning approaches have tremendously scaled up AI-based cancer management in several aspects. Although genetics and imaging have not been combined explicitly for bladder cancer staging, AI-based radiogenomic analysis has increased the accuracy in the diagnosis, prediction, and staging of many other cancers, such as breast and lung cancer [21,22,23]. Taken together, we hypothesize that the state-of-the-art processing techniques and analysis tools associated with radiogenomics can be the foundation for a sophisticated staging system to more accurately stage bladder cancers. Therefore, in this study, we (1) identified genetic signatures that significantly help characterize the stages of bladder cancer, (2) analyzed the morphological and textural properties of the bladder tumors in magnetic resonance (MR) scans to seek out unique features that are not appreciated by the human eye but can potentially assist in identifying the stage of cancer, and (3) developed an automated system that integrates both genetic and MR features and characterizes the bladder cancer as intra-vesical (T1 and T2), tumors still stay within the bladder vs. extra-vesical (T3 and T4), and tumors have grown through the muscle layer of the bladder and into the layer of fatty tissue.

## 2. Results

### 2.1. Identification of Genomics Features

**Figure 1:**
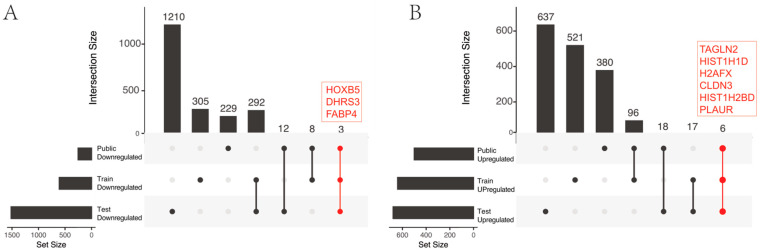
Intersection analysis of differential genes across three datasets. The upset plot illustrates the intersecting differential genes from train, test, and public databases. (**A**) represents the intersecting downregulated genes when comparing high- and low-stage samples. (**B**) demonstrates the intersecting upregulated genes when comparing high- and low-stage samples. The red color and dots symbolize the intersecting differential genes derived from the train, test, and public databases.

The analysis revealed nine common genes, three of which exhibited higher expression levels in low-stage cases (lower expression levels in high stage): HOXB5, DHRS3, and FABP4, whereas six had higher expression levels in high-stage cases (lower expression levels in low stage): TAGLN2, HIST1H1D, HIST1H2BD, H2AFX, CLDN3, and PLAUR, as shown in Figure 1.

**Figure 2:**
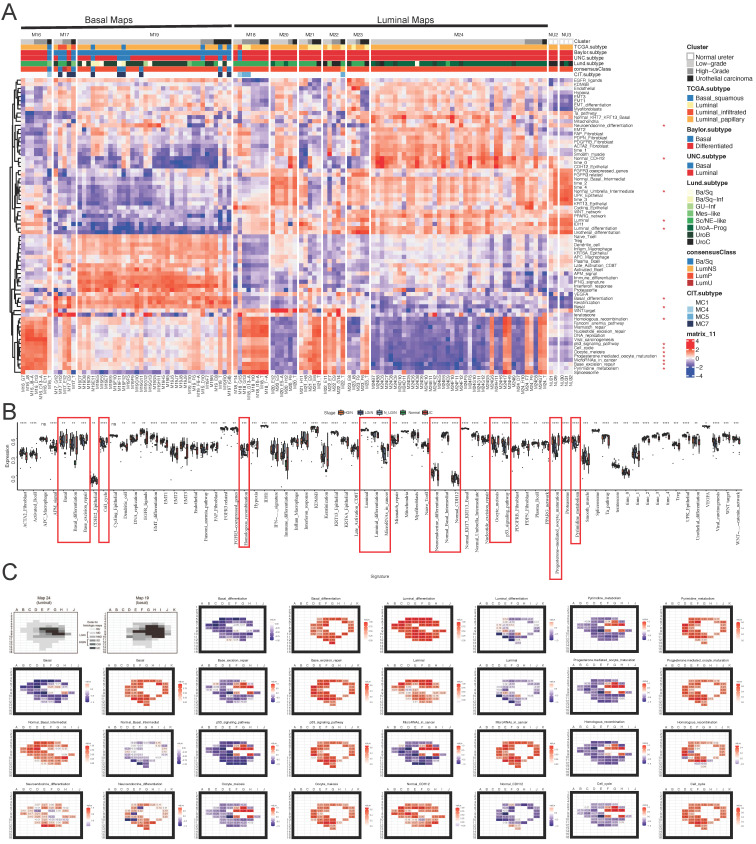
(**A**) Luminal, Luminal_differentiation, Neuroendocrine_differentiation, Normal_Basal_Intermediate, and Normal_CDH12 signatures, Basal, Basal_differentiation, Base_excision_repair, Cell_cycle, Homologous_recombination, MicroRNAs_in_cancer, Oocyte_meiosis, p53_signaling_pathway, Progesterone-mediated_oocyte_maturation, Proteasome, and Pyrimidine_metabolism. (**B**) List of significant signatures: Basal, Basal_differentiation, Base_excision_repair, Cell_cycle, Homologous_recombination, MicroRNAs_in_cancer, Oocyte_meiosis, p53_signaling_pathway, Progesterone-mediated_oocyte_maturation, Proteasome, and Pyrimidine_metabolism. The red frame accentuates 15 significant signatures associated with different stages of bladder cancer. *: *p* < 0.05, **: *p* < 0.01, ***: *p* < 0.001, ****: *p* < 0.0001 and ‘ns’: not significant. (**C**): NU, normal urothelium; MD, mild dysplasia; MdD, moderate dysplasia; SD, severe dysplasia; CIS, carcinoma in situ; UC, urothelial carcinoma. For analytical purposes, samples of MD and MdD were combined and referred to as LGIN. Samples of SD and CIS were combined and referred to as HGIN, normal urothelium (NU), and low- and high-grade intra-urothelial neoplasia (LGIN and HGIN).

Based on the analysis using the single-sample Gene Set Enrichment Analysis (ssGSEA) algorithm, we successfully identified and corroborated 15 significant signatures related to the various stages of bladder cancer (as shown in Figure 2A). Among the identified signatures, some exhibited a decreasing trend from NU (normal urothelium) to UC (urothelial carcinoma), with the highest expression in normal urothelium and progressively decreasing through mild, moderate, and severe dysplasia stages and carcinoma in situ. These signatures were Luminal, Luminal differentiation, Neuroendocrine differentiation, Normal Basal Intermediate, and Normal CDH12. In contrast, other signatures demonstrated the opposite trend, being lowest in normal urothelium and increasing as dysplasia severity increased, reaching their peak in urothelial carcinoma, as given in Figure 2B. The results from these analyses were subsequently confirmed using comprehensive histological and genomic mapping data from two patients (one basal and one luminal), as shown in Figure 2C. The findings obtained using *Data_K_* were validated using *Data_W_* and *Data_B_*. The statistical significance test was performed on *Data_W_* and *Data_B_*, where the identified 9 and 15 genes were found to be significantly different in intra- vs. extra-vesical bladder cancer.

### 2.2. Identification of Radiomics Features

**Figure 3:**
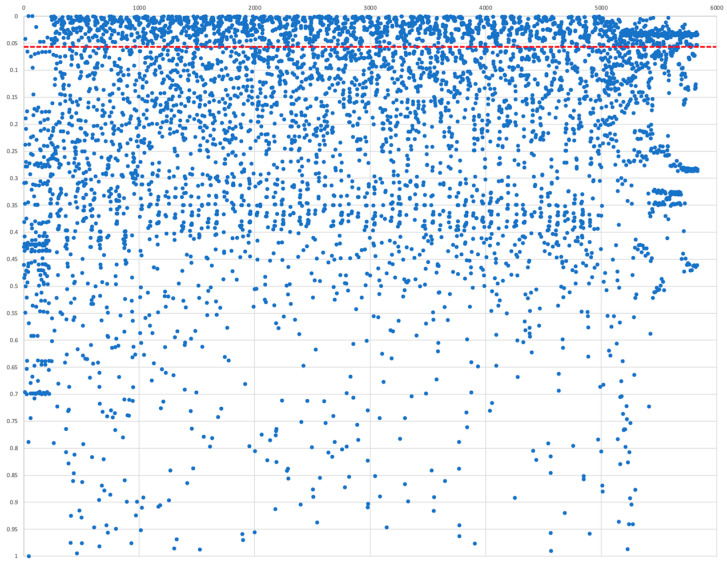
The Manhattan plot shows the distribution of *p*-values obtained for each of the MRI-based radiomic features in comparison to low- and high-stage bladder cancer. The datapoints over the solid red line correspond to features with significance at 5% (i.e., *p* < 0.05).

Several statistical tests were performed on the extracted features from all 28 images of *Data_K_* using different analysis tools, including R, Matlab, and SPSS. Three major tasks were completed in the following order: (1) obtain a set of clean and uniform features (e.g., by eliminating nonzero or inoperable values), (2) reduce the redundancy of the feature sets, and (3) identify the most significant features in terms of the statistical difference between extra- and intra-vesical tumors. The statistical Student’s *t*-test and Bhattacharya coefficient were performed on the filtered set of features to identify the significantly different features between the two groups. About 33% of the total number of extracted radiomic features showed significance at a *p*-value of 0.05. The findings supported the primary hypothesis, as the analysis identified MR features distinguishing extra-vesical and intra-vesical bladder tumors. The Manhattan plot in Figure 3 is a pictorial description of the *p*-values obtained for all the usable features. Most of the identified significant features were texture-based.

### 2.3. Integration of Radiomics and Genomics Features Improves Bladder Cancer Staging

The performance of the proposed radiogenomic model for automated bladder cancer staging was evaluated by considering the staging as a binary classification problem where one MIBC case categorized as a false stage is considered a failure. The model was evaluated using three matrices, including sensitivity (true positive rate, i.e., TP/P), specificity (true negative rate, i.e., TN/N), and accuracy, i.e., (TP + TN)/(P + N). For the current evaluation, P (positive) referred to extra-vesical bladder cancer, whereas N (negative) referred to intra-vesical bladder cancer. Also, the model was designed to maximize sensitivity, as it is clinically more important to identify cases if they have already turned from an intra-vesical (early stage) to an extra-vesical (late stage) bladder tumor. In other words, a false-positive (intra-vesical bladder tumor wrongly classified as an extra-vesical bladder tumor) is relatively more manageable than a false-negative (extra-vesical tumor wrongly identified as an intra-vesical tumor).

The radiogenomic staging model (RGs) was trained on all 28 cases of *Data_K_* using the method explained in the previous section. The external validation was performed using all 12 test cases of *Data_N_*. During external validation, the model used the same set of genetic and radiomic predictors identified in the training phase. The five machine learning classifiers generated satisfactory results, whereas the NB generated the highest mean classification sensitivity, specificity, and accuracy, reaching up to 94%, 88%, and 92%, respectively. The three matrices calculated for the training and testing of five classifiers are given in Table 1. The existing clinical staging method results in a sensitivity of 53.3%, a specificity of 87.5%, and an accuracy of 71.0%. The results suggest that RGs show more accurate staging performance than the existing clinical staging method.

Two separate bladder staging models, one using genomic features (G_S_) and the other using radiomic features (R_S_), were trained and tested. The same five classifiers (Naïve Bayes, NB; Support Vector Machine, SVM; K-Nearest Neighbor, KNN; Logistic Regression, LR; and Decision Tree, DT) were trained for each of the genomic (G_S_) and radiomic (R_S_) models in conjunction with the RFE method. The significant features identified in the initial genomic and radiomic analysis using *Data_K_* were used for the training of G_S_ and R_S_, with a limit of up to 8 maximum features for each model. The G_S_ model was tested using the genomic features in the three external models, that is, *Data_N_*, *Data_W_*, and *Data_B_*, where the SVM obtained the highest mean classification sensitivity, specificity, and accuracy at 77%, 55%, and 67%, respectively. The RS model was tested using the MR images in *Data_N_*, where the NB obtained the highest mean classification sensitivity, specificity, and accuracy at 77%, 72%, and 75%, respectively. The results show that the RGs model takes the lead by 25% and 17% from the G_S_ and R_S_ models, respectively, on mean classification accuracy, which supports the primary hypothesis of the study that integrating genomic and radiomic features can improve the staging accuracy of bladder cancer more than genomic or radiomic analysis alone.

All scripts for the described analysis were designed at the host institute and implemented on Matlab version 2023a. During external validation, all features extracted from previously unseen MR images lay within the expected range of values, and no infinity or undefined values were encountered. The model remained stable, and no thread crashed at any step throughout the analysis.

## 3. Discussion

Currently, the clinical staging system for bladder cancer is based on the results of the transurethral resection of the bladder tumor/exam under anesthesia and imaging tests, specifically axial imaging of the abdomen and pelvis with CT-based imaging exams in the United States. As per the National Comprehensive Cancer Network (NCCN) guidelines, CT urography is recommended for patients suspected of bladder cancer, providing a detailed evaluation of the bladder, lymph nodes, potential metastases, and upper tract disease before TURBT [24]. Two common techniques for CT urography are the single-bolus and split-bolus techniques, each with its advantages and considerations. While cystoscopy remains the gold standard for bladder evaluation, CT is widely used for the detection and staging of bladder urothelial carcinoma. CT urography, involving unenhanced, urothelial, and excretory phases, is valuable for both upper tract and bladder assessment, aiding in staging and post-treatment follow-up [24]. The sensitivity and specificity of CT urography for detecting bladder UC are reported to be as high as 93% and 99% [25,26,27], making it particularly useful for identifying invasive tumors. Interestingly, in Japan, bladder cancer patients undergo both a CT scan to assess for nodal involvement/distant metastasis and an MRI scan to assess the bladder for specific T-stage or intra- or extra-vesical bladder cancer.

It is believed that MRI possesses better resolution than a CT scan and thus could be more accurate in staging bladder cancer [28]. Furthermore, the inclusion of genomic features into MRI imaging holds the potential to greatly enhance bladder cancer staging, which could lead to improved medical decision-making. This is the first study showing the possibility that the automated integration of genetic expressions and MR features can efficiently assist in identifying the accurate stage of bladder cancer. While the naïve approach to distinguishing tumors of a low stage from a high stage primarily relies on tumor size, in this study, the textural and shape properties of the low- and high-stage bladder tumors (that apparently may have identical sizes) were thoroughly analyzed through radiomics to identify dissimilar features that are usually overlooked or uninterpretable by the naked eye. Several robust machine learning classifiers were deployed to explore the optimal combination of genetic and radiomic predictors to obtain the highest staging accuracy. Although the NB outperformed, the performances of other classifiers on three matrices were still comparable, showing the consistency of the identified significant features in genomic and radiomic analysis. A part of the reason for the enhanced performance of NB could be its probabilistic framework, effectively managing uncertainty in predictors from multiple omics, especially when dealing with limited training data. Moreover, the classification accuracy of the proposed radiogenomic model was on average 25% and 17% higher than that of exclusive genomic- and radiomic-based models, respectively.

To conduct these analyses, multiple retrospective datasets of gene expressions and MR scans of bladder cancer patients were collected. First, an extensive investigation of gene expressions and radiomic features of MR scans of bladder cancer patients was performed. The analysis identified several potential predictors specific to MIBC in both genetic and MR data, which validated our primary hypothesis. The analysis was followed by the development of a radiogenomic model for MIBC staging in which several machine learning algorithms were trained to perform automated binary classification of bladder cancer cases into either a low- or high-stage group using different combinations of the newly identified genetic and MR features. These algorithms were then evaluated at different performance bars to identify the one with the highest classification accuracy on the training data. The selected model was then validated against an independent external dataset, and the performance was estimated using different matrices. Moreover, two additional models, one based on genetic features and the other based on MR features alone, were trained and tested for MIBC staging. The performance of the proposed radiogenomic model was found to be significantly higher than the other two models, which strongly supports our proof of concept. This first study has high clinical application and encourages further investigation by replicating the model and validating its performance on large datasets.

The study’s goal is to assist clinicians in better exploring and deciding optimal treatment, given a certain stage of cancer, to improve the outcome. The clinical stages associated with tumor growth, however, may be different, as stages Ta, Tis, and T1 are known as non-muscle-invasive bladder cancer (NMIBC), while stages T2–T4 are known as MIBC [29]. However, the current study categorizes these stages into two major classes, that is, intra-vesical (Ta, Tis, and T1) and extra-vesical (T2, T3, and T4), based on the complexity of the subsequent treatment. For instance, patients at stage Ta or T1 (low grade) are primarily treated with TURBT, followed by immediate intra-vesical chemotherapy. These patients are prone to developing more low-stage tumors throughout their lives [30]. Similarly, stage Ta, T1 (high grade), or Tis is commonly treated with a combination of TURBT and intra-vesical Bacillus Calmette–Guerin (BCG) immunotherapy or chemotherapy [24,31]. It has been observed that such tumors may return at a more advanced stage [24]. Patients at T2 or higher are given more aggressive and urgent treatment, including highly invasive surgeries. As we discussed, past studies have shown that the outcomes of bladder preservation with chemoradiotherapy are survival outcomes comparable to those of radical cystectomy when the patients suitable for the bladder-preserving strategy were properly selected, that is, not patients with T3 or T4 bladder cancer [6,8,9]. Our study demonstrates improved clinical staging of bladder cancer patients when a novel automated radiogenomic model is utilized. Such an automated radiogenomic model can be easily deployed in a clinical setting. Patients with a suspected bladder tumor seen on cystoscopy could undergo axial imaging of the pelvis with an MR scan (with contrast and T2 weighted images), followed by transurethral resection of the bladder tumor and RNA-seq or RT-PCR of a prescribed RNA signature. Subsequently, the automated radiogenomic model would more accurately stage bladder cancer patients, leading to improved outcomes.

Our study has three key contributions. First, a new set of genetic expressions potentially predictive of low and high stages of bladder cancer was identified that includes Luminal, Luminal differentiation, Neuroendocrine differentiation, Normal Basal Intermediate, and Normal CDH12. Second, it is the first time that the microlevel irregularities in the bladder tumors were investigated using radiomics of MR images, with the goal of uncovering patterns of tumor texture associated with the stage of bladder cancer. Various MR features potentially predictive of bladder cancer stages were identified, suggesting that imaging can play an essential role in characterizing stages of MIBC. Third, the first automated radiogenomic model for bladder cancer staging was developed and validated on an independent dataset. The model outperformed the two models, each developed based on genomic and radiomic features alone. The RFE method, in conjunction with several common machine learning classifiers, efficiently identified the most optimal combination of radiomic and genomic features for accurate staging.

One of the study’s strengths lies in the utilization of AI models, which systematically integrate complex genomic and radiomic data to characterize key features influencing disease progression, enabling accurate staging of bladder cancer. The increasing interest in employing AI for radiogenomics in various cancer-related objectives has been noted [32], owing to its superior performance that surpasses nearly all manual approaches. AI has proven instrumental in correlating radiomic patterns with specific genomic signatures across distinct cancer stages. For example, Gillies et al. [33] demonstrated the potential of radiomics in predicting the mutation status of isocitrate dehydrogenase in gliomas. Specifically, each of the five models investigated has been commonly employed in radiogenomic models for various cancers [34]. While these models refine existing staging criteria and offer a more individualized assessment of a patient’s disease status, challenges such as the need for larger datasets for model training and validation, the interpretability of AI-generated features, and the careful integration of these findings into clinical decision-making processes need consideration.

It is important to note that the model training prioritized maintaining higher sensitivity compared to specificity. Sensitivity, in this context, refers to accurately classifying cases into the high-stage category. This decision was intentional to minimize ‘false-negative’ instances (incorrectly identifying a ‘high-stage’ tumor as a ‘low-stage’ tumor). Justifying this choice ensures that cases classified as ‘low stage’ but actually ‘high stage’ (false-positive) undergo further evaluation, which may not have as severe consequences as the scenario where a ‘high stage’ case is incorrectly classified as ‘low stage’ (false-negative), potentially preventing the timely intervention to stop cancer growth to the advanced stage, that is, ‘high stage’.

### Limitations and Future Work

The key limitation in this study lies in the scarcity of available datasets for analysis. However, given that the main purpose of this initial analysis is to establish a proof of concept and present the potential of the idea through preliminary findings, the existing data adequately serves the investigative goals. Subsequent validation of the proposed model on a more extensive dataset would not only affirm our findings but also refine the model parameters, resulting in a more robust staging system for MIBC.

Another constraint of the study is that it does not attempt to establish associations between the integrated, identified features and the underlying biological mechanisms. The biological interpretation of radiomic features, such as tumor texture, poses significant challenges. A comprehensive analysis involving a substantial number of cases and accompanying histopathological information could offer an opportunity to correlate predictive features with the underlying biology, thereby enhancing our understanding.

An important avenue for improving the model’s accuracy is to integrate demographic and clinical characteristics that could significantly impact the overall staging of bladder cancer. However, there is a risk of overfitting, and it would be challenging to discern the fundamental contribution of specific features to the staging performance, potentially undermining the primary goal of investigating the combined clinical value of radiomics and genomics. For future studies with larger datasets, the inclusion of demographic or clinical characteristics could enhance the stability and overall performance of bladder cancer staging.

## 4. Materials and Methods

### 4.1. Study Data

The study was performed after approval by the Cedars-Sinai Medical Center Institutional Review Board (IRB) (STUDY00001310), Kyoto University IRB (#G1301), and Nara Medical University IRB (#2967) under a request for waiver of consent on archived pathologic specimens and magnetic resonance imaging (MRI). Potential study subjects were identified by searching for both MRI and pathology data repositories from both Japanese centers for the years between 2010 and 2020. Each case in both datasets came with a deidentified imaging report specifying the clinical stage of the cancer and the final pathologic stage obtained at the time of radical cystectomy. Next, we gathered a comprehensive collection of RNA and MRI data and bladder tumor formalin-fixed paraffin-embedded (FFPE) at the time of TURBT from these two centers, where MRI imaging of the bladder is routinely performed for staging bladder cancer, as shown in Table 1, with one center serving as a training cohort and the other as a testing cohort. The training cohort consisted of 28 cases from the Kyoto University Hospital (termed *Data_K_*), including both MRI scans and RNA sequencing (RNA-seq). The cases belong to 25 males and 3 females, with a median age of 78. Among these cases, 18 were classified as intra-vesicle tumor cases (Ta, Tis, T1, and T2), while the remaining 10 cases were categorized as extra-vesicle tumor cases (T3, T4, and N+ or M+). The testing cohort consisted of 12 cases from Nara Medical University Hospital (termed *Data_N_*), including both MRI scans and RNA sequencing (RNA-seq). The cases belong to 11 males and 1 female, with a median age of 71. Out of these cases, 6 were classified as intra-vesicle tumor cases, while the remaining 6 cases were identified as extra-vesicle tumor cases. The subjects in both datasets belong to Asian ethnic groups.

Moreover, two public datasets, one published by Wrana JL. et al. in Eur Urol in 2014 (termed *Data_W_*) [35] and the other by Jolanta Bondaruk et al. in iScience in 2022 (termed *Data_B_*) [36], were also included in the study. The *Data_W_* consists of the next-generation RNA-seq of archival formalin-fixed paraffin-embedded urothelial bladder cancer. The dataset consisted of 27 cases categorized as intra-vesicle tumor cases and 22 cases categorized as extra-vesicle tumor cases, whereas the *Data_B_* consists of geographically annotated mucosal samples from human cystectomies performed in 9 patients with bladder cancer (3 basal and 6 luminal molecular subtypes) and 2 subjects with normal bladders. The samples ranged from normal urothelium (NU) to urothelial carcinoma (UC), obtained from individual patients, exhibiting varying degrees of dysplasia, including mild dysplasia, moderate dysplasia, severe dysplasia, and carcinoma in situ. These samples comprised microscopically normal urothelium, in situ preneoplastic conditions known as low-stage intra-urothelial neoplasia and high-grade intra-urothelial neoplasia, as well as UC. By incorporating these diverse datasets, the aim of the study was to provide a comprehensive and multi-dimensional understanding of bladder cancer at different clinical stages, leveraging RNA sequencing and MRI data to gain insights into the disease’s underlying mechanisms and potential biomarkers.

As a part of preprocessing, the RNA isolation was performed on FFPE curls using the FormaPure XL RNA isolation kit (Beckman Coulter, Brea, CA, USA). Purified total RNA was tested for purity using the NanoDrop 8000 (ThermoFisher Scientific, Waltham, MA, USA), quantified using the Qubit Flex fluorometer (ThermoFisher Scientific), and the 2100 Bioanalyzer (Agilent Technologies, Santa Clara, CA, USA). Also, the library construction was performed using the Lexogen QuantSeq 3′ mRNA-Seq Library Prep Kit FWD for Illumina. (Lexogen, Vienna, Austria). Briefly, all RNA samples were assessed for concentration using a Qubit fluorometer and for quality using the 2100 Bioanalyzer. Up to 100 ng of total RNA per sample was used for library preparation. Library concentration was measured with a Qubit fluorometer, and library size was measured on an Agilent 4200 TapeStation (Agilent Technologies). Libraries were multiplexed and sequenced on a NovaSeq 6000 (Illumina, San Diego, CA, USA) using 75 bp single-end sequencing. On average, about 10 million reads were generated from each sample.

### 4.2. Radiogenomic Staging of Bladder Cancer

The following sections describe an automated methodology for radiogenomic-based staging of bladder cancers.

#### 4.2.1. Genomic Analysis of MIBC Staging

The purpose of the genomic analysis of patients with bladder cancer was to identify gene expressions that are significantly different between intra- and extra-vesicle tumors. For this, RNA-seq technology was employed to capture a comprehensive snapshot of the transcriptome. To ensure data reliability and accuracy, stringent quality control measures were implemented throughout the RNA-seq workflow. The data were ensured to be the most accurate by carefully evaluating the quality of the sequencing reads, assessing library complexity, and monitoring various metrics.

A differential analysis was conducted using the DESeq2 package in R to compare gene expression between low- and high-stage samples in the *Data_K_*. DESeq2 employs empirical Bayes methods to accurately identify differentially expressed genes (DEGs) from count data. By analyzing DEGs, we gain insights into the molecular changes associated with disease progression and potential therapeutic targets or biomarkers. By overlapping the differentially expressed genes, we were able to categorize them separately based on their higher expression in intra- vs. extra-vesical cases.

To assess the relevance of these gene signatures, the ssGSEA algorithm was applied. The ssGSEA algorithm is a widely used computational method that quantifies the activity of predefined gene sets in individual samples. It measures the enrichment score of each gene set in each sample by comparing the ranks of the genes in the set to the ranks of all other genes in the sample. This approach enables us to evaluate the activity levels of the BLCA (Bladder Urothelial Carcinoma) stage-related gene signatures in each sample, providing insights into the underlying biology and potential clinical implications. By applying the ssGSEA algorithm to *Data_K_*, we assessed the enrichment of the bladder cancer-stage-related gene signatures in different stages of bladder cancer. This analysis helped identify the gene sets that are significantly associated with specific stages of bladder cancer. Our focus was on a set of 75 bladder cancer-stage-related signatures (identified in multiple published references [12,37,38]), with the goal of identifying and validating the most significant signatures while considering potential confounding effects stemming from patient-to-patient variability.

#### 4.2.2. Radiomic Analysis of Bladder Cancer Staging

Radiomic analysis is a rapidly growing approach to extracting and thoroughly analyzing the high-throughput image features of anatomical structures, including tumors, to assist in prediction, diagnosis, and prognosis. Using predefined mathematical quantities, radiomics can characterize tumor phenotypes based on complex multidimensional arrays of image-derived measurements. Radiomic analysis has been used for staging lung, gastric, cervical, and rectal cancers [39,40,41,42].

An extensive radiomic analysis of bladder tumors using MR scans was performed to seek features that are significantly different between intra- vs. extra-vesical tumors. The analysis was performed using all 28 high-resolution T2-weighted MR scans in *Data_K_*. Preprocessing included outlining the tumors in all MR images by two independent and experienced radiologists. Any labeling disagreements were resolved through consensus with a third radiologist. The ITK-snap version 3.0 software was used throughout the interactive labeling process. Each of the 28 images in *Data_K_* was normalized (i.e., voxel values in each image were scaled between 0 and 1) before analysis using the min-max scaling. Several thousand radiomic features were then extracted from the outlined bladder tumors in each of these images. Each radiomic feature represented a unique MR image characteristic of the tumor and was expressed as a single numerical value calculated using a standard pre-defined mathematical formula. Various radiomic features were considered, including 15 First Order Statistics (e.g., Kurtosis, Percentiles, Range, etc.), 20 Gray-level Co-occurrence Matrix statistics (e.g., Cluster shade, Contrast, Autocorrelation, etc.), 15 Gray Level Run Length Matrix statistics (e.g., Run percentage, Run entropy, etc.), 14 Gray Level Size Zone Matrix (e.g., Zone percentage, Zone variance, etc.), 12 Gray Level Dependence Matrix (e.g., Small dependence emphasis, etc.), 20 Shape-based Features 2D/3D (e.g., Volume, Surface area, Sphericity, etc.), and 5 others (e.g., Complexity, Busyness, etc.). For example, to extract the signal intensity of the bladder tumor region, the mean gray level values of all voxels in the outlined boundary of the tumor in all slices of a three-dimensional bladder MR scan were considered.

All radiomic features were extracted using different combinations of three significantly important radiomic parameters, including bin size, kernel size, and angle. The Bin size determines the number of bins in the discretization process of the scan. The discretization decreases the chance of noise amplitude by transferring the continuous values of voxels into discrete counterparts, avoiding contrast variation among all MR scans acquired from different scanners. The Kernel size specifies the proximity around a voxel as a fixed window within which the spatial relationships of voxels with each other are calculated. The Angle determines the directions in which the spatial neighborhood is to be considered within the Kernel window. The choice of the three parameters has a great influence on the overall analysis. The Bin size used during feature extraction was between 2x = 1 and 2x = 8. The window size for the Kernel could take values between 1 and 5, whereas the Angle in all four quadrants was considered. Thus, an exhaustive analysis was performed using all possible combinations of the three parameters, which yielded nearly 6000 radiomic features from each of the 28 images in *Data_K_*. All features were extracted using an in-house radiomic feature-extraction application.

#### 4.2.3. Radiogenomic Modeling for Bladder Cancer Staging

A machine learning model was developed that performs automated staging of bladder cancer through the integration of the above genomic and radiomic predictors. A systematic approach was adopted to ensure that the proposed model selects the minimal set of reliable and consistent predictors from both data categories to avoid overfitting and increasing generalizability without compromising the overall performance of the model.

The preprocessing steps include identifying a subset of the significant features identified in the radiomic analysis that show the highest predictive strength. Such features were obtained in two phases: the first phase consisted of randomly selecting one of the multiple features that differed only in the combination of the radiomic parameters these were obtained with. For example, if multiple instances of a feature (e.g., contrast) were identified as significant (each with a unique combination of Bin size, Kernel size, and Angle), then only one of the instances would be selected and the rest would be ignored. In the second phase, the subset of features was further narrowed to a sub-subset by choosing the features that show significance at a minimal *p*-value such that the number of features in the final set corresponds to the number of features identified as significant in the genomic analysis. Figure 3 presents the distribution of *p*-values for all the features examined and those that show significance at 0.05. The final set of radiomic features consisted of 12 unique features identified at *p* = 0.01, as Energy, Inverse gaussian left, Inverse gaussian left focus, Gaussian right polar, Cluster promin, Cluster shade, Cluster trend, Homogeneity, Autocorrelation, Gaussian, Gaussian right focus, and Gaussian right polar.

Subsequently, several common fully supervised machine learning classifiers were trained to perform automated staging of bladder cancer using both sets of significant features. The staging was expressed as a binary classification problem, with intra- vs. extra-vesical as two possible categories. The NB classifier worked based on the Bayes theorem with the “naive” assumption of conditional independence between every pair of features (e.g., feature independence within and across radiomic and genetic feature sets). The KNN performed classification using the assumption of the proximity of values for features from a class (e.g., values of radiomic features from low-stage tumors were expected to be closer than those from high-stage tumors). The SVM performed classification by specifying among hyperplanes and separating the radiomic and genomic features of two classes such that the margins of the planes are maximized. The LR worked based on the linear regression technique, which finds an optimal boundary between two sets of features using a logistic sigmoid function to avoid outliers. The DT classifier worked by specifying nodes (each denoting a radiomic/genomic feature), branches (each denoting a decision of the test), and terminal nodes (each representing one of the two stages of bladder cancer).

These algorithms were chosen for analysis due to their widespread acceptance in binary classification problems and frequent utilization for their performance. These algorithms have been employed in numerous studies to identify the critical aspects of patients’ conditions and model the progression of diseases following treatment using intricate health information and medical datasets [34]. These algorithms have demonstrated stability in addressing cancer staging problems, including recurrence of stage IV colorectal cancer [43], cancer genomics and subtyping of breast cancer [44], and detection of early stages of pancreatic cancer [18]. Moreover, given the pilot nature of this study and the small dataset, we steered clear of advanced or complex algorithms to prevent potential overfitting.

Finally, the Recursive Feature Elimination (RFE) method was deployed in conjunction with the five mentioned classifiers to eliminate the relatively weak features by comparing the overall training accuracy achieved by each classifier using different combinations of features. A classifier could select up to a maximum of 8 significant features from each of two categories (i.e., genomics and radiomics) while maximizing the classification accuracy. Any features from each of the two categories were counted with equal weight.

### 4.3. Statistical Analysis

Statistical analyses were performed with R version 4.0.3 (https://www.r-project.org/, accessed on 1 August 2021), and the visualization of heat maps was achieved using the Complex Heat Maps Bioconductor package. To assess differences among multiple groups for continuous variables, Kruskal–Wallis tests were applied. Additionally, the Gene Set Enrichment Analysis of tumor single-cell subtype signatures was conducted using the single sample Gene Set Enrichment Analysis (ssGSEA) function from the R package GSVA. All *p*-values were two-sided, and *p* < 0.05 was considered to indicate a statistically significant difference.

## 5. Conclusions

This preliminary study provides a proof-of-concept with promising results of a radiogenomic approach for bladder cancer staging and encourages researchers to further validate the proposed model on large datasets. Although the number of cases in the study data was low, the methodology ensured avoiding overfitting by restricting the number of predictors used in the model. Furthermore, with the certainty that at least 40% of the enrolled subjects are from each of the low- and high-stage groups, a relatively small number of cases was sufficient to develop the classification model. The lack of specific and well-established tumor characteristics to recognize its stage leads to inefficient management of bladder cancer. To our knowledge, the proposed data structure has not been used previously for bladder cancer staging. The future works include performing an exhaustive search on data repositories at the host and the collaborating centers to further validate the model on large datasets and improve its generalizability. This will also allow for the establishment of a correlation between imaging indicators and genetic heterogeneity. Taken together, a robust model can be a supporting tool in prospective studies and will help increase the rate of efficient bladder cancer staging.

## Figures and Tables

**Table 1:** The results of the binary classification of bladder cancer cases into low- and high-stage cancer using a radiogenomic model. Five classifiers were tested on external data *Data_N_*.

Classifier	Validation (*Data*_N_)		
	Sen.	Spe.	Acc.
NB	0.94	0.88	0.92
SVM	0.88	0.78	0.83
DT	0.89	0.72	0.80
KNN	0.78	0.72	0.75
LR	0.72	0.78	0.75

## Data Availability

The data presented in this study are available on request from the corresponding author. The data are not publicly available due to the upcoming follow up manuscript.

## References

[1] Siegel R.L., Miller K.D., Wagle N.S., Jemal A. (2023). Cancer statistics, 2023. CA Cancer J. Clin..

[2] Howlader N., Noone A.M., Krapcho M., Garshell J., Neyman N., Altekruse S.F., Kosary C.L., Yu M., Ruhl J., Tatalovich Z. SEER Cancer Statistics Review, 1975–2010. http://seer.cancer.gov/csr/1975_2010/.

[3] Brausi M., Witjes J.A., Lamm D., Persad R., Palou J., Colombel M., Buckley R., Soloway M., Akaza H., Bohle A. (2011). A review of current guidelines and best practice recommendations for the management of nonmuscle invasive bladder cancer by the International Bladder Cancer Group. J. Urol..

[4] Stenzl A., Cowan N.C., De Santis M., Kuczyk M.A., Merseburger A.S., Ribal M.J., Sherif A., Witjes J.A., European Association of Urology (2011). Treatment of muscle-invasive and metastatic bladder cancer: Update of the EAU guidelines. Eur. Urol..

[5] Calabro F., Sternberg C.N. (2012). Metastatic bladder cancer: Anything new?. Curr. Opin. Support. Palliat. Care.

[6] Stein J.P., Lieskovsky G., Cote R., Groshen S., Feng A.C., Boyd S., Skinner E., Bochner B., Thangathurai D., Mikhail M. (2001). Radical cystectomy in the treatment of invasive bladder cancer: Long-term results in 1054 patients. J. Clin. Oncol..

[7] Dhar N.B., Klein E.A., Reuther A.M., Thalmann G.N., Madersbacher S., Studer U.E. (2008). Outcome after radical cystectomy with limited or extended pelvic lymph node dissection. J. Urol..

[8] Efstathiou J.A., Spiegel D.Y., Shipley W.U., Heney N.M., Kaufman D.S., Niemierko A., Coen J.J., Skowronski R.Y., Paly J.J., McGovern F.J. (2012). Long-term outcomes of selective bladder preservation by combined-modality therapy for invasive bladder cancer: The MGH experience. Eur. Urol..

[9] Mak R.H., Hunt D., Shipley W.U., Efstathiou J.A., Tester W.J., Hagan M.P., Kaufman D.S., Heney N.M., Zietman A.L. (2014). Long-term outcomes in patients with muscle-invasive bladder cancer after selective bladder-preserving combined-modality therapy: A pooled analysis of Radiation Therapy Oncology Group protocols 8802, 8903, 9506, 9706, 9906, and 0233. J. Clin. Oncol..

[10] Nepple K.G., O’Donnell M.A. (2009). The optimal management of T1 high-grade bladder cancer. Can. Urol. Assoc. J..

[11] Batista R., Vinagre N., Meireles S., Vinagre J., Prazeres H., Leao R., Maximo V., Soares P. (2020). Biomarkers for Bladder Cancer Diagnosis and Surveillance: A Comprehensive Review. Diagnostics.

[12] Kamoun A., de Reynies A., Allory Y., Sjodahl G., Robertson A.G., Seiler R., Hoadley K.A., Groeneveld C.S., Al-Ahmadie H., Choi W. (2020). A Consensus Molecular Classification of Muscle-invasive Bladder Cancer. Eur. Urol..

[13] Chamie K., Ballon-Landa E., Bassett J.C., Daskivich T.J., Leventhal M., Deapen D., Litwin M.S. (2015). Quality of diagnostic staging in patients with bladder cancer: A process-outcomes link. Cancer.

[14] Ozaydin S., Atas E., Karadurmus N., Emirzeoglu L., Arpaci F. (2020). Outcomes of bladder preservation therapy on survival in patients with muscle-invasive bladder cancer. Arch. Esp. Urol..

[15] Wang H., Luo C., Zhang F., Guan J., Li S., Yao H., Chen J., Luo J., Chen L., Guo Y. (2019). Multiparametric MRI for Bladder Cancer: Validation of VI-RADS for the Detection of Detrusor Muscle Invasion. Radiology.

[16] Trivizakis E., Papadakis G.Z., Souglakos I., Papanikolaou N., Koumakis L., Spandidos D.A., Tsatsakis A., Karantanas A.H., Marias K. (2020). Artificial intelligence radiogenomics for advancing precision and effectiveness in oncologic care (Review). Int. J. Oncol..

[17] Saxena S., Jena B., Gupta N., Das S., Sarmah D., Bhattacharya P., Nath T., Paul S., Fouda M.M., Kalra M. (2022). Role of Artificial Intelligence in Radiogenomics for Cancers in the Era of Precision Medicine. Cancers.

[18] Qureshi T.A., Gaddam S., Wachsman A.M., Wang L., Azab L., Asadpour V., Chen W., Xie Y., Wu B., Pandol S.J. (2022). Predicting pancreatic ductal adenocarcinoma using artificial intelligence analysis of pre-diagnostic computed tomography images. Cancer Biomark..

[19] Mahajan A., Sahu A., Ashtekar R., Kulkarni T., Shukla S., Agarwal U., Bhattacharya K. (2023). Glioma radiogenomics and artificial intelligence: Road to precision cancer medicine. Clin. Radiol..

[20] Ninatti G., Kirienko M., Neri E., Sollini M., Chiti A. (2020). Imaging-Based Prediction of Molecular Therapy Targets in NSCLC by Radiogenomics and AI Approaches: A Systematic Review. Diagnostics.

[21] Perez-Johnston R., Araujo-Filho J.A., Connolly J.G., Caso R., Whiting K., Tan K.S., Zhou J., Gibbs P., Rekhtman N., Ginsberg M.S. (2022). CT-based Radiogenomic Analysis of Clinical Stage I Lung Adenocarcinoma with Histopathologic Features and Oncologic Outcomes. Radiology.

[22] Wang Y., Wang Y., Guo C., Xie X., Liang S., Zhang R., Pang W., Huang L. (2020). Cancer genotypes prediction and associations analysis from imaging phenotypes: A survey on radiogenomics. Biomark. Med..

[23] Gallivanone F., Bertoli G., Porro D. (2022). Radiogenomics, Breast Cancer Diagnosis and Characterization: Current Status and Future Directions. Methods Protoc..

[24] Flaig T.W., Spiess P.E., Agarwal N., Bangs R., Boorjian S.A., Buyyounouski M.K., Chang S., Downs T.M., Efstathiou J.A., Friedlander T. (2020). Bladder Cancer, Version 3.2020, NCCN Clinical Practice Guidelines in Oncology. J. Natl. Compr. Canc Netw..

[25] Trinh T.W., Glazer D.I., Sadow C.A., Sahni V.A., Geller N.L., Silverman S.G. (2018). Bladder cancer diagnosis with CT urography: Test characteristics and reasons for false-positive and false-negative results. Abdom. Radiol..

[26] Lee C.H., Tan C.H., Faria S.C., Kundra V. (2017). Role of Imaging in the Local Staging of Urothelial Carcinoma of the Bladder. AJR Am. J. Roentgenol..

[27] Sadow C.A., Silverman S.G., O’Leary M.P., Signorovitch J.E. (2008). Bladder cancer detection with CT urography in an Academic Medical Center. Radiology.

[28] Barentsz J.O., Jager G.J., Witjes J.A., Ruijs J.H. (1996). Primary staging of urinary bladder carcinoma: The role of MRI and a comparison with CT. Eur. Radiol..

[29] National Cancer Institute Bladder Cancer Stages. https://www.cancer.gov/types/bladder/stages.

[30] Aldousari S., Kassouf W. (2010). Update on the management of non-muscle invasive bladder cancer. Can. Urol. Assoc. J..

[31] Babjuk M., Bohle A., Burger M., Capoun O., Cohen D., Comperat E.M., Hernandez V., Kaasinen E., Palou J., Roupret M. (2017). EAU Guidelines on Non-Muscle-invasive Urothelial Carcinoma of the Bladder: Update 2016. Eur. Urol..

[32] Aerts H.J., Velazquez E.R., Leijenaar R.T., Parmar C., Grossmann P., Carvalho S., Bussink J., Monshouwer R., Haibe-Kains B., Rietveld D. (2014). Decoding tumour phenotype by noninvasive imaging using a quantitative radiomics approach. Nat. Commun..

[33] Gillies R.J., Kinahan P.E., Hricak H. (2016). Radiomics: Images Are More than Pictures, They Are Data. Radiology.

[34] Kang J., Rancati T., Lee S., Oh J.H., Kerns S.L., Scott J.G., Schwartz R., Kim S., Rosenstein B.S. (2018). Machine Learning and Radiogenomics: Lessons Learned and Future Directions. Front. Oncol..

[35] Liu Y., Noon A.P., Aguiar Cabeza E., Shen J., Kuk C., Ilczynski C., Ni R., Sukhu B., Chan K., Barbosa-Morais N.L. (2014). Next-generation RNA sequencing of archival formalin-fixed paraffin-embedded urothelial bladder cancer. Eur. Urol..

[36] Bondaruk J., Jaksik R., Wang Z., Cogdell D., Lee S., Chen Y., Dinh K.N., Majewski T., Zhang L., Cao S. (2022). The origin of bladder cancer from mucosal field effects. iScience.

[37] Robertson A.G., Kim J., Al-Ahmadie H., Bellmunt J., Guo G., Cherniack A.D., Hinoue T., Laird P.W., Hoadley K.A., Akbani R. (2017). Comprehensive Molecular Characterization of Muscle-Invasive Bladder Cancer. Cell.

[38] Gouin K.H., Ing N., Plummer J.T., Rosser C.J., Ben Cheikh B., Oh C., Chen S.S., Chan K.S., Furuya H., Tourtellotte W.G. (2021). An N-Cadherin 2 expressing epithelial cell subpopulation predicts response to surgery, chemotherapy and immunotherapy in bladder cancer. Nat. Commun..

[39] Umutlu L., Nensa F., Demircioglu A., Antoch G., Herrmann K., Forsting M., Grueneisen J.S. (2020). Radiomics Analysis of Multiparametric PET/MRI for N- and M-Staging in Patients with Primary Cervical Cancer. Rofo.

[40] Liu Q., Li J., Xin B., Sun Y., Feng D., Fulham M.J., Wang X., Song S. (2021). (18)F-FDG PET/CT Radiomics for Preoperative Prediction of Lymph Node Metastases and Nodal Staging in Gastric Cancer. Front. Oncol..

[41] Lin X., Zhao S., Jiang H., Jia F., Wang G., He B., Jiang H., Ma X., Li J., Shi Z. (2021). A radiomics-based nomogram for preoperative T staging prediction of rectal cancer. Abdom. Radiol..

[42] Huang Y., Liu Z., He L., Chen X., Pan D., Ma Z., Liang C., Tian J., Liang C. (2016). Radiomics Signature: A Potential Biomarker for the Prediction of Disease-Free Survival in Early-Stage (I or II) Non-Small Cell Lung Cancer. Radiology.

[43] Xu Y., Ju L., Tong J., Zhou C.M., Yang J.J. (2020). Machine Learning Algorithms for Predicting the Recurrence of Stage IV Colorectal Cancer After Tumor Resection. Sci. Rep..

[44] Cascianelli S., Molineris I., Isella C., Masseroli M., Medico E. (2020). Machine learning for RNA sequencing-based intrinsic subtyping of breast cancer. Sci. Rep..

